# Molecular evidence for ten species and Oligo-Miocene vicariance within a nominal Australian gecko species (*Crenadactylus ocellatus*, Diplodactylidae)

**DOI:** 10.1186/1471-2148-10-386

**Published:** 2010-12-15

**Authors:** Paul M Oliver, Mark Adams, Paul Doughty

**Affiliations:** 1 Australian Centre for Evolutionary Biology and Biodiversity, The University of Adelaide, Darling Building, Adelaide SA 5005, Australia; 2 South Australian Museum, Adelaide, SA 5000, Australia; 3 Evolutionary Biology, South Australian Museum, Adelaide, SA 5000, Australia; 4 Terrestrial Zoology, Western Australian Museum, 49 Kew St, Welshpool WA 6106, Australia

## Abstract

**Background:**

Molecular studies have revealed that many putative 'species' are actually complexes of multiple morphologically conservative, but genetically divergent 'cryptic species'. In extreme cases processes such as non-adaptive diversification (speciation without divergent selection) could mask the existence of ancient lineages as divergent as ecologically and morphologically diverse radiations recognised as genera or even families in related groups. The identification of such ancient, but cryptic, lineages has important ramifications for conservation, biogeography and evolutionary biology. Herein, we use an integrated multilocus genetic dataset (allozymes, mtDNA and nuclear DNA) to test whether disjunct populations of the widespread nominal Australian gecko species *Crenadactylus ocellatus *include distinct evolutionary lineages (species), and to examine the timing of diversification among these populations.

**Results:**

We identify at least 10 deeply divergent lineages within the single recognised species *Crenadactylus ocellatus*, including a radiation of five endemic to the Kimberley region of north-west Australia, and at least four known from areas of less than 100 km^2^. Lineages restricted to geographically isolated ranges and semi-arid areas across central and western Australia are estimated to have began to diversify in the late Oligocene/early Miocence (~20-30 mya), concurrent with, or even pre-dating, radiations of many iconic, broadly sympatric and much more species-rich Australian vertebrate families (e.g. venomous snakes, dragon lizards and kangaroos).

**Conclusions:**

Instead of a single species, *Crenadactylus *is a surprisingly speciose and ancient vertebrate radiation. Based on their deep divergence and no evidence of recent gene flow, we recognise each of the 10 main lineages as candidate species. Molecular dating indicates that the genus includes some of the oldest vertebrate lineages confounded within a single species yet identified by molecular assessments of diversity. Highly divergent allopatric lineages are restricted to putative refugia across arid and semi-arid Australia, and provide important evidence towards understanding the history and spread of the Australian arid zone, suggesting at a minimum that semi-arid conditions were present by the early Miocene, and that severe aridity was widespread by the mid to late Miocene. In addition to documenting a remarkable instance of underestimation of vertebrate species diversity in a developed country, these results suggest that increasing integration of molecular dating techniques into cryptic species delimitation will reveal further instances where taxonomic conservatism has led to profound underestimation of not only species numbers, but also highly significant phylogenetic diversity and evolutionary history.

## Background

Whereas traditional field and morphological studies continue to discover new species [1], complexes of phenotypically similar unrecognised taxa are now increasingly identified through molecular systematic examination of 'known' taxa [2-4]. Documenting this wealth of 'cryptic species' (two or more morphologically similar, but not necessarily identical, species confounded within one) is a priority of modern systematic research [5]. All species, however, are not equal: their phylogenetic distinctiveness (i.e. evolutionary distance from nearest living relatives) can vary enormously [6-8]. Many clades are characterised by relative morphological stasis over very long time periods [9]; within such groups, 'cryptic species' might be divergent lineages as ancient as ecologically diverse nominal 'genera' or even 'families' of more morphologically variable clades [9,10]. Identifying such ancient cryptic diversity is likely to provide important insights into biogeographic history and processes of morphological stasis, and is essential for the effective allocation of conservation resources to preserve the maximal breadth of evolutionary diversity [5]. Nonetheless, even though the techniques are readily available, cryptic species assessments have not systematically integrated techniques such as internally calibrated molecular dating to assess the phylogenetic diversity [6,7] of newly identified taxa.

Pygopodoid (formerly diplodactyloid or diplodactylid) geckos are a Gondwanan radiation of lizards restricted to Australia and surrounding islands [11,12]. A recent molecular phylogenetic study of the pygopodoids, found the monotypic genus *Crenadactylus *to be among the most divergent extant lineages [12]. The single nominal species in the genus, *Crenadactylus ocellatus *is a secretive scansorial lizard, Australia's smallest gecko species (<39 mm snout-vent length), and broadly distributed across isolated patches in the west, centre and north of Australia [13]. Two papers have examined the taxonomy of this species over the last three decades and four subspecies are now recognised [14,15]. A more recent molecular study revealed very deep genetic divergences between these nominal subspecies [12]; and at least one recognised subspecies (*C. o. horni*) also spans multiple widely isolated and disjunct biogeographic regions [13], suggesting the genus may harbour additional species level diversity.

*Crenadactylus *are rarely collected over much of their range, many northern populations are known from very few sites and poorly represented in museum collections, and it is only through recent extensive fieldwork that sufficient samples have become available for a comprehensive genetic analysis. In this study we used independent mitochondrial (*ND2*) and nuclear (*RAG-1*, *C-mos*, allozymes) loci to estimate specific and phylogenetic diversity within the nominal species '*Crenadactylus ocellatus*' from localities spanning its wide range across arid and semi-arid Australia. Populations for which there was congruent evidence of lack of gene flow and historical independence (fixed allozyme differences and relatively high mtDNA divergence and monophyly) were regarded to represent candidate species (see methodology outlined in detail elsewhere [4]). This new sampling and data revealed a striking instance of severe underestimation of phylogenetic diversity, with important ramifications for conservation and understanding the environmental history of Australia.

## Results

### Species diversity and distributions

**Figure 1:**
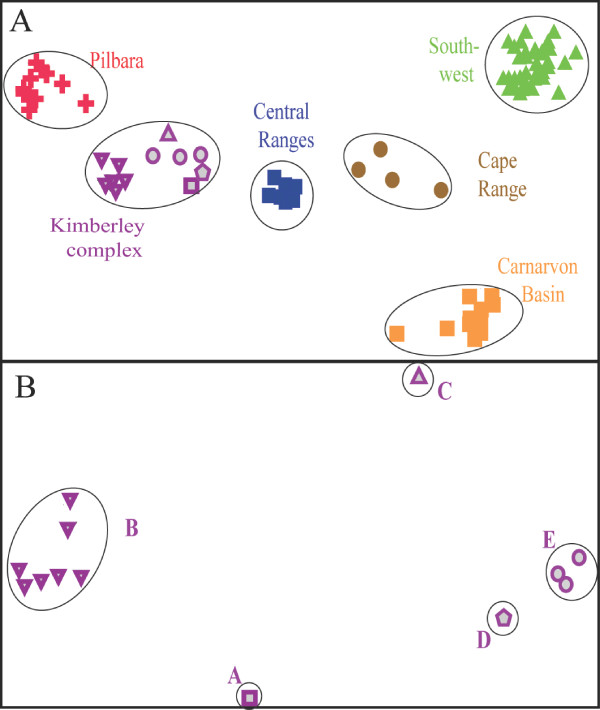
**Allozyme data for *Crenadactylus***. Selected Principal Co-ordinates Analyses, based on the allozyme data. The relative PCO scores have been plotted for the first (X-axis) and second (Y-axis) dimensions. (*A*) PCO of all 94 *Crenadactylus *sampled. The first and second PCO dimensions individually explained 30% and 16% respectively of the total multivariate variation. (*B*) PCO of the 13 Kimberley *Crenadactylus*. The first and second PCO dimensions individually explained 51% and 11% respectively of the total multivariate variation.

An initial Principal Co-ordinates Analysis (PCO) of allozyme data for all 94 individuals (Figure 1A) revealed the presence of six primary clusters, one for each of six different geographic regions: South West, Carnarvon Basin, Cape Range, Pilbara, Kimberley, and Central Ranges. Each cluster was diagnosable from all others by 6-19 fixed differences, supporting their status as distinct taxonomic entities (Table 1). Follow-up PCOs on each cluster found only modest within-group heterogeneity (i.e. no obvious subgroups, or subgroups differing by less than three fixed differences) in all but one regional cluster, namely that representing the Kimberley specimens. Here, PCO identified five genetically distinctive subgroups (Kimberley A-E; Figure 1B), each differing from one another by 4-14 fixed differences (Table 1), and all differentiated by fixed differences involving "private" alleles at one or more of the loci (range = 1-4 loci; Table S1). A final round of PCOs on subgroups Kimberley B and Kimberley E (the only two Kimberley lineages represented by more than one specimen) did not reveal any obvious genetic subdivision.

**Table 1:** Allozyme summary.

Taxon	1	2	3	4	5	6	7	8	9	10
**1. South-West**	-	0.304	0.355	0.619	0.560	0.657	0.640	0.542	0.623	0.447
**2. Carnarvon Basin**	10 (24%)	-	0.446	0.794	0.572	0.728	0.810	0.597	0.740	0.531
**3. Cape Range**	9 (21%)	13 (31%)	-	0.700	0.593	0.557	0.629	0.591	0.607	0.637
**4. Pilbara**	18 (43%)	21 (50%)	20 (48%)	-	0.439	0.386	0.479	0.491	0.498	0.574
**5. Kimberley A**	18 (44%)	18 (44%)	17 (41%)	13 (32%)	-	0.194	0.404	0.253	0.314	0.534
**6. Kimberley B**	20 (48%)	22 (52%)	15 (36%)	13 (31%)	7 (17%)	-	0.368	0.414	0.435	0.593
**7. Kimberley C**	20 (48%)	22 (52%)	18 (43%)	16 (38%)	14 (34%)	11 (26%)	-	0.321	0.262	0.689
**8. Kimberley D**	16 (39%)	17 (41%)	17 (41%)	16 (39%)	9 (22%)	13 (32%)	12 (29%)	-	0.120	0.525
**9. Kimberley E**	18 (43%)	21 (50%)	16 (38%)	16 (38%)	10 (24%)	12 (29%)	8 (19%)	4 (10%)	-	0.575
**10. Central Ranges**	15 (36%)	17 (40%)	17 (40%)	16 (38%)	16 (39%)	19 (45%)	20 (48%)	16 (39%)	17 (40%)	-

Matrix of pairwise genetic distances from allozyme data among 10 candidate species of *Crenadactylus*. Lower left triangle = number of fixed differences (%FD in brackets); upper right triangle = unbiased Nei D.

**Figure 2:**
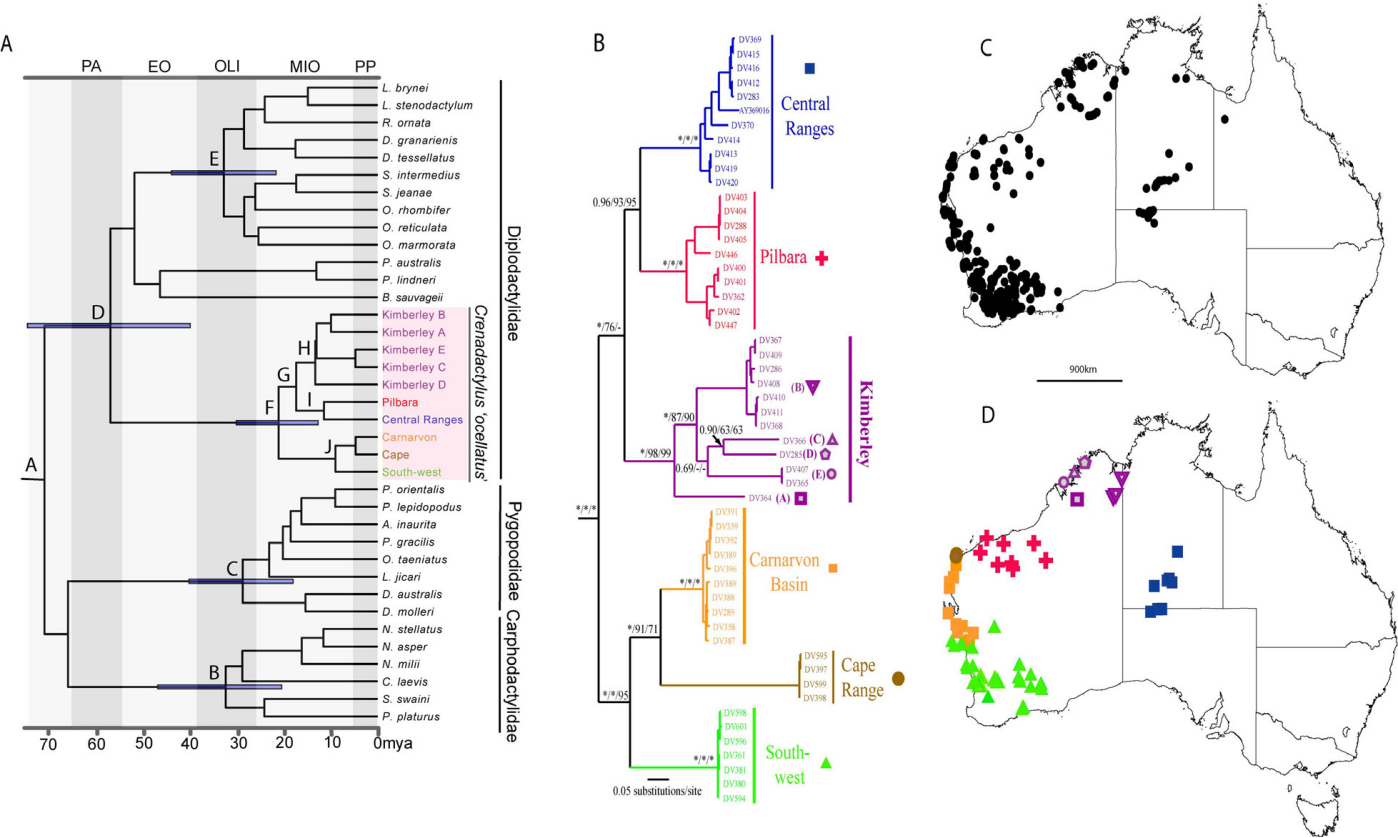
**Phylogeny and distribution of *Crenadactylus***. (*A*) Bayesian chronogram showing estimated age of 10 candidate species of *Crenadactylus *and exemplars of major lineages of pygopodoids based on concatenated nuclear dataset. Letters at major nodes correspond with those in Table 1. (*B*) Bayesian consensus tree from *ND2 *data showing structure and relationships between 10 candidate taxa of *Crenadactylus *with Bayesian, ML and MP support values for key nodes (values of 1.00 or 100 indicated by*). (*C*) Known localities of *Crenadactylus *based on voucher specimens in all Australian Museums. (*D*) Localities and nominal taxonomic designation for each genetically typed specimen included in our analyses.

Bayesian and maximum likelihood phylogenetic analyses of nuclear and mitochondrial data identified these same 10 groups as deeply divergent lineages (Additional file 1, Table 2), and reciprocally monophyletic where multiple samples were available (Figure 2A, B). Minimum corrected and uncorrected pairwise (mitochondrial) genetic divergences between candidate species (>22.1/15.3%) were much higher than maximum distances within candidate species (<11.6/9.7%) (see Table 2 and Additional file 1, Table S2, respectively), further emphasising their long periods of historical isolation.

**Table 2:** Mitochondrial divergences.

	N	1	2	3	4	5	6	7	8	9	10
**1. South-west**	7	_	0.235	0.183	0.212	0.219	0.256	0.217	0.245	0.239	0.222
**2. Cape Range**	4	0.623	_	0.201	0.246	0.254	0.289	0.255	0.264	0.262	0.267
**3. Carnarvon**	10	0.359	0.456	_	0.205	0.205	0.243	0.220	0.240	0.232	0.225
**4. Pilbara**	10	0.505	0.709	0.494	_	0.171	0.206	0.192	0.209	0.199	0.185
**5. Central Ranges**	11	0.512	0.718	0.457	0.294	_	0.203	0.202	0.221	0.217	0.207
**6. Kimberley A**	1	0.704	1.059	0.673	0.445	0.415	_	0.191	0.174	0.153	0.153
**7. Kimberley B**	1	0.557	0.872	0.577	0.421	0.443	0.347	_	0.181	0.174	0.153
**8. Kimberley C**	2	0.657	0.923	0.698	0.470	0.491	0.281	0.333	_	0.161	0.165
**9. Kimberley D**	1	0.680	0.981	0.686	0.463	0.504	0.221	0.326	0.266	_	0.139
**10. Kimberley E**	7	0.615	0.997	0.636	0.405	0.479	0.248	0.281	0.281	0.227	_

Corrected (GTR+I+G) and uncorrected genetic distances between ten candidate species confounded within '*Crenadactylus ocellatus*', calculated using 828 bp of ND2 data.

Based on both independent and combined analysis of mitochondrial and nuclear sequence data (Figure 2, Additional file 2) the basal dichotomy within *Crenadactylus *was between a south/western clade (three major lineages) and a north/central clade (seven major lineages). The south/western clade included three parapatric lineages, two endemic to the Cape Range area and Carnarvon coast respectively, and a more deeply divergent lineage widespread throughout the southwest of Western Australia. The north/central clade comprised an endemic radiation of five allopatric lineages from the Kimberley (northern Western Australia), and a pair of sister taxa from the Pilbara region and the Central Ranges (Figures 2B, D). Allopatric populations within the north/central clade are largely restricted to rocky ranges and showed high levels of geographically structured mtDNA diversity, while the two widespread taxa in the south/western clade were not restricted to rocky ranges, and were characterised by very low levels of mtDNA divergence across their distribution, suggestive of significant recent gene flow or range expansion (Additional file 1, Table 2).

### Divergence dating and age of cryptic radiation

Topology and node support for the pygopodoid phylogeny recovered by the dating analyses was consistent across nuclear and combined datasets, and with similar datasets presented elsewhere [12]. The 95% height intervals for all age estimates were relatively wide (Table 3), due to our explicit incorporation of calibration error. Using the estimated age of *Crenadactylus *from the nuclear and combined analysis as secondary prior, the 95% CI for the estimated mean rate of mitochondrial sequence evolution per lineage per million years within *Crenadactylus *was between 0.96-2.24% (nuclear calibrations) to 0.72-1.76% (combined calibrations), broadly consistent with published estimates of rates from other squamate groups (0.47-1.32% per lineage per million years) [16].

**Table 3:** Bayesian age estimates.

	nuclear	combined	combined no 3rds
Posteriors			
Outgroups			
Root	113.9 (82.7-145.2)	113.3 (81.5-142.8)	114.5 (84.3-145.7)
(A) Pygopodoidea	69.3 (51.0-89.4)	65.4 (47.0-83.6)	67 (48.0-85.1)
(B) Carphodactylidae	31.5 (19.9-36.7)	39.7 (27.2-54.5)	36.7 (23.9-50.3)
(C) Pygopodidae	28 (17.5-39.2)	28.2 (19.2-38.2)	26.2 (17.2-35.6)
(D) Diplodactylidae	55.6 (38.9-72.9)	56.2 (40.8-73.3)	56.4 (39.2-72.8)
(E) Core Diplodactylidae	32 (21.0-42.9)	37.1 (26.5-49.4)	34.8 (23.2-46.4)
*Crenadactylus*			
(F) Crown	20.5 (12.3-29.3)	31.5 (21.7-41.9)	30.7 (20.6-41.4)
(G) Northern	16.9 (9.9-24.0)	27 (18.5-36.4)	25.9 (17.2-35.6)
(H) Kimberley	12.9 (7.1-19.3)	19.9 (13.3-27.3)	18.2 (11.5-25.4)
(I) Pilbara/Central Ranges	11.1 (4.3-17.3)	21 (13.0-30.0)	18.8 (9.8-27.9)
(J) Southern	8.7 (3.4-14.5)	23.1 (15.2-32.2)	21.5 (13.2-30.7)
Calibrations			
Root	uniform 80-150	uniform 80-150	uniform 80-150
Pygopodoidea	normal 71.5 (12.5)	normal 71.5 (12.5)	normal 71.5 (12.5)

Comparison of mean and range (95% posterior density distribution) of divergence time estimates for selected outgroup and *Crenadactylus *nodes based on Bayesian dating analyses (BEAST) of three different sets of alignment data. Age estimates are in millions of years and letters alongside major splits correspond with labels in Fig. 2A.

Actual and relative age estimates for the four major clades of pygopodoids (C, D, E, F (see methods)) were broadly similar (Figure 2A, Table 3). However, the estimated age of crown *Crenadactylus*, and the relative age of this radiation in comparison with the other three major Australian pygopodoid gecko radiations was significantly older when using combined data as opposed the nuclear data alone (Table 3). We suggest that the combination of shallow calibration points and saturation of the mitochondrial component of the combined data, and/or stochastic error given the relatively few substitutions in the nuclear dataset may explain this discrepancy. The older dates from combined datasets are viewed as a potential maximum while the younger dates from the nuclear data are viewed as a conservative minimum. Nuclear data suggest that the initial diversification of crown *Crenadactylus *occurred in the late Oligocene to early Miocene (10-30 million years ago (mya)), and that it is probably slightly younger, but nonetheless broadly concurrent with diversification in the other three major Australian clades of Pygopodoidea (Table 3). If the combined analysis is more correct than the nuclear only analysis, it would indicate that crown *Crenadactylus *is significantly older (i.e. late Oligocene 20-40 mya). Both datasets indicate that the four major geographic isolates of *Crenadactylus *(Western/South-west, Central Ranges, Pilbara and Kimberley) had all diverged by the late Miocene, approximately 10 mya.

## Discussion

### Cryptic species diversity and conservation

**Figure 3:**
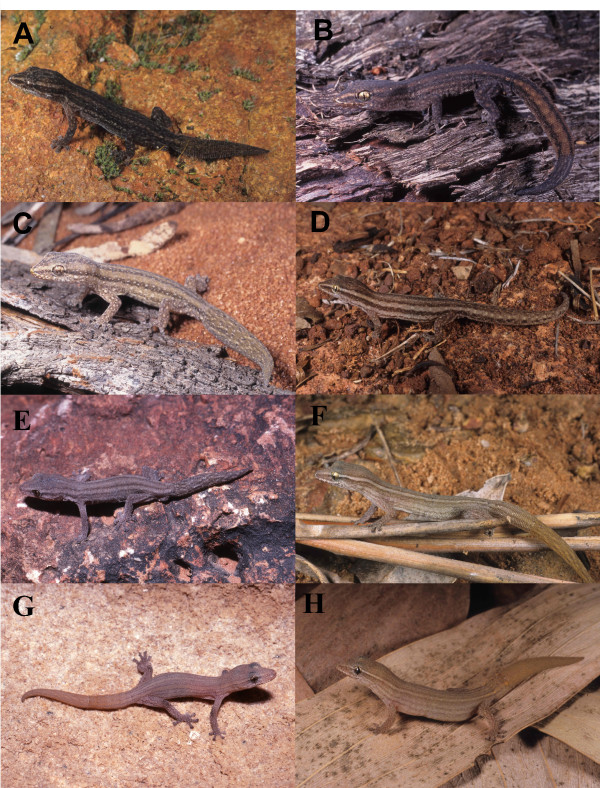
**Candidate species of *Crenadactylus***. Pictures in life of 8 of the 10 candidate species currently confounded within the nominal species *Crenadactylus ocellatus *A) South-west B) Carnarvon C) Cape Range D) Pilbara E) Central Ranges F) Kimberley B G) Kimberley D and H) Kimberley E. Photos courtesy Brad Maryan, Glenn Gaikhorst, Glenn Shea.

Based on the high levels of uncorrected mtDNA divergence (>15%; even higher if corrected), multiple fixed allozyme differences, reciprocal mtDNA monophyly and deep divergence times we estimate that at least 10 lineages of *Crenadactylus *are evolutionarily divergent, non-interbreeding and warrant recognition as candidate species (exemplars of eight of these lineages photographed in life are illustrated in Figure 3). Many of these lineages are further defined by multiple nuclear differences. A full taxonomic revision of the genus is currently in preparation. Thereafter, *Crenadactylus ocellatus *will be restricted to the south-west population as the type locality is in the Perth area, the three other recognised subspecies will be elevated to full species, and additional new species will be described. Ongoing analysis and published data also suggest that at least some of these taxa are morphologically diagnosable on the basis of subtle features of scalation and colouration (Doughty and Oliver pers. obs.).

Our estimate of total species diversity is almost certainly conservative for several reasons. At least five candidate species are potential short-range endemics [17] (Cape Range, Kimberley A, C, D, and E): thus, *Crenadactylus *lineages have clearly persisted and speciated in relatively small patches of suitable habitat. This would indicate that known and geographically isolated, but genetically unsampled, populations of *Crenadactylus *from the Kimberley and around the Queensland/Northern Territory border (Figure 2C) may include additional unrecognised taxa. *Crenadactylus *are secretive, rare, and difficult to collect (for example four of the five Kimberley taxa were each represented by only a single site in this study), and as large areas across northern and central Australia have not been intensively surveyed, it seems likely that additional populations (potential species) remain undetected. Finally, maximum levels of genetic diversity within the Central Ranges and Pilbara candidate species are moderately high (7.9-9.7% uncorrected), and further work may reveal that these candidate species each comprise complexes of multiple cryptic taxa.

The identification of a clade of five candidate species within the Kimberley region of north-west Australia is also notable. Whereas morphological work has identified micro-endemic allopatric radiations of species within some Kimberley invertebrate lineages [18], this is the first genetic evidence for moderately extensive *in situ *speciation within the region, and the only documented evidence of a moderately diverse (>3 species) endemic vertebrate radiation. Few other areas of similar size within Australia contain comparably diverse endemic vertebrate radiations (examples include the wet tropics (microhylid frogs: *Cophixalus*) and Tasmania (skinks: *Niveoscincus*)) [19,20]. The results of this and a growing body of other work emphasise the biogeographic importance, environmental complexity, high endemism and phylogenetic diversity of the rugged and poorly known Kimberley [21].

Most candidate species of *Crenadactylus *are from areas of low human impact however, restricted range taxa with potentially narrow climatic tolerances are particularly vulnerable to rapid anthropogenic climate change [22]. The diversity we have uncovered within *Crenadactylus *underlines how an overly conservative taxonomy and patchy sampling may obscure the existence of range-restricted taxa at potentially high risk of extinction. Northern Australia remains relatively poorly sampled, and ongoing studies indicate it probably represents one of the largest remaining frontier areas for modern systematic research and inventory in a developed country [4,23]. In light of unprecedented global environmental changes and the apparently high levels of endemism within this area, systematic surveys and genetic assessments of diversity to address this oversight should be a high priority. If not, there is a risk that many deeply divergent, but morphologically conservative lineages will disappear before they are even documented.

### Divergence dates

Based on our secondary calibrations, the initial diversification of lineages currently confounded within '*Crenadactylus ocellatus*' probably began in the mid to early Miocene (~20 mya) and potentially at least 10 million years earlier. One recognised subspecies of *Crenadactylus *(*C. o. horni*) includes four candidate species (Carnarvon, Cape Range, Pilbara, and Central Ranges) that span the basal divergence of the genus (Figure 2A, B). None of these lineages have been formally recognised or named and they satisfy the definition for cryptic species we are following herein. Given that rigorous molecular dating with direct calibrations is rarely integrated into assessment of cryptic species diversity, it remains to be seen how common such deeply divergent cryptic lineages are. However, the divergence times between these unrecognised species of *Crenadactylus *are amongst the oldest documented for any cryptic species of tetrapod, and comparable with the oldest cryptic species identified in vertebrates [24], subterranean amphipods [25], and perhaps exceeded only by copepods [10]. In contrast to these studies, our date estimates are also based on internally calibrated trees, as opposed to generalised and often unreliable global estimates for rates of sequence evolution [26].

The depth of divergences among candidate species of *Crenadactylus *(and within other pygopodoid genera such as *Diplodactylus*, *Lucasium *and *Salturius *[27-29]) are comparable with vertebrate radiations noted for their extreme morphological conservatism (e.g. *Plethodon *salamanders) [30]. The long-term conservatism of these cryptic radiations of geckos is particularly striking in light of the major environmental changes they have persisted through (see below) and the great morphological plasticity of related lineages such as the legless lizards (Pygopodidae) [15].

Our date estimates indicate the extensive evolutionary diversity hidden within the nominal species '*Crenadactylus ocellatus*' is as ancient as ecologically diverse crown radiations of many iconic endemic Australian groups: terrestrial venomous snakes (~10 mya, 102+ spp, 26 genera) [31], agamid lizards (~23 mya, 71+ spp, 13 genera) [32], most macropods (~20 mya, 70+ spp, 14 genera) [33] and murine rodents (early Pliocene, ~5 mya 160+ spp, 31 genera) [34]. Likewise, while allozyme data cannot provide reliable divergence dates [35], levels of allozyme genetic divergence found within *Crenadactylus *(mean = 36.7%FD, range 10-52%FD; Additional file 1, Table S1) are similar to the entire Australian radiation (17 spp, 5 genera) [15] of 'short-necked' chelid turtles (mean 35.8%FD, range = 0-57%FD) [36]. In contrast to the single recognised 'species' of *Crenadactylus *these are all broadly co-distributed radiations of Australasian vertebrates that are widespread across biomes, include multiple named genera or even families, and show extensive sympatry and ecological diversity across major lineages currently afforded generic or higher rank.

Analysis of nuclear and combined datasets further indicate that initial divergence of the ancestral *Crenadactylus *lineage from other pygopodoids (as opposed to the crown radiation of extant lineages within this genus) was broadly contemporaneous with, or even pre-dated, initial diversification of iconic Gondwanan Australasian clades such as the basal oscine birds [37], most major Australasian marsupial families [38], pelodryad treefrogs [39], many major lineages of the Proteaceae [40], and *Nothofagus *[41]. The divergence of the *Crenadactylus *lineage from other extant geckos also pre-dates current estimates for the initial radiation of most other extant squamate (lizard and snake) families in Australia by at least 10-20 million years [12]. The only comparably divergent Australian squamate genus identified to date are the cave geckos (*Pseudothecadactylus*), however this lineage appears to be (at least distantly) related to an extralimital radiation of geckos in New Caledonia [12]. Thus, *Crenadactylus *is not only unexpectedly diverse, but also the only surviving representative of a relatively ancient lineage. Indeed current evidence indicates that it is the most phylogenetically divergent endemic genus in the diverse Australian squamate fauna of over 870 spp and 115 genera [15].

### Ancient vicars across the Australian arid zone

*Crenadactylus *are among Australia's smallest terrestrial vertebrates. While small body size increases vulnerability to environmental conditions, it also allows access to micro-refugia inaccessible to larger vertebrates [42]. As with other lineages showing outward morphological conservatism over long timescales, an absence of major morphological differentiation since the early Miocene also suggests a relatively constrained ecology [9]. Each of the four major geographic clusters of *Crenadactylus *sampled (Kimberley, Central Ranges, Pilbara and South-west/Carnarvon/Cape Range) are allopatric and restricted to relatively temperate semi-arid or rocky areas, separated by expanses of arid desert. These geographically isolated and deeply divergent lineages of *Crenadactylus *appear to be relatively ancient relics of a former much wider distribution, now greatly attenuated by the expansion of severe aridity.

Dated phylogenies for many major Australian vertebrate and faunal radiations are now available, and all generally indicate the fauna of the arid zone (the largest biome in Australia and one of the largest arid landforms in the world) is the result of a complete turnover since the estimated onset of aridification around 20 mya, and that most endemic lineages are significantly younger than 20 mya [43]. Thus far *Crenadactylus *is the only vertebrate lineage showing strong evidence for a contrasting pattern; the persistence of multiple lineages that pre-date the estimated onset of severe aridification in refugia, both around and within the arid zone. Indeed, they are currently the oldest known allopatric sister lineages of Australian vertebrates restricted to isolated ranges and relatively mesic coastal pockets through the semiarid to arid west, centre and north of Australia.

*Crenadactylus *thus spans both the geographic extent and temporal origins of the arid zone, but does not seem to have adapted to it. Like the relatively few other ancient relict lineages present (e.g stygobiontic beetles) [44], this pattern provides potentially important insights into the spread of aridity. In this case, the timing of diversification of *Crenadactylus *lineages supports the suggestion that semi-arid/seasonally arid conditions (to which the lineage is restricted) date back to at least the mid-Miocene (the basal split within the crown radiation), and that severe aridity dates back to the late Miocene (the oldest splits between multiple major lineages which are now geographically isolated by very arid desert). Age estimates for the separation of multiple, geographically-isolated candidate species in *Crenadactylus *also provide perhaps the strongest phylogenetic support yet for the hypothesis that significantly arid conditions were already widespread across west and central Australia in the 'Hill gap', 6-10 mya [43], a period where depositional records are poor, making it difficult to assess historical Australian climates.

## Conclusion

Our data have revealed that the single nominal species *'Crenadactylus ocellatus' *comprises a moderately diverse and surprisingly ancient complex of numerous unrecognised and highly divergent lineages. The distribution and antiquity of these lineages suggests that with further work incorporating additional sampling, ecological analysis, physiological data and environmental niche modelling, *Crenadactylus *will be an important evolutionary radiation for understanding the deep history of arid Australia. More generally, integration of data and techniques from diverse fields into the delimitation of species boundaries is a growing focus of taxonomic work (integrative taxonomy [45,46]). Our results demonstrate how integration of molecular dating techniques into cryptic species analysis can quantify the depth of phylogenetic divergences and reveal patterns of great evolutionary interest and conservation significance within lineages showing outward morphological conservatism.

## Methods

### Sampling

Ninety-five *Crenadactylus *specimens were sampled for genetic analysis. Allozyme profiles were successfully scored for 94 individuals and a representative subset of these (N = 53) were sequenced for the *ND2 *gene (Additional file 1, Table S3). Based on the results of mitochondrial and allozyme analysis, we obtained nuclear data (*RAG-1*) for exemplars of the ten most divergent lineages of *Crenadactylus*. For dating analyses we also incorporated published *C-mos *data for three representative deep lineages spanning crown *Crenadactylus *[12]. Outgroups (Additional file 1, Table S4) were selected from published diplodactylid, carphodactylid, pygopodid, gekkonid and sphaerodactylid sequences on GenBank [12].

### Allozyme analyses

Allozyme analyses of liver homogenates were undertaken on cellulose acetate gels according to established procedures [47]. The final allozyme dataset (Additional file 1, Table S1) consisted of 94 *Crenadactylus *genotyped at 42 putative loci. The following enzymes displayed banding patterns of sufficient activity and resolution to permit allozymic interpretation: ACON, ACP, ACYC, ADH, AK, DIA, ENOL, EST, FDP, FUM, GAPD, GLO, GOT, GPD, GPI, GSR, IDH, LAP, LDH, MDH, MPI, NDPK, NTAK, PEPA, PEPB, PGAM, 6PGD, PGK, PGM, SOD, SORDH, TPI, and UGPP. Details of enzyme/locus abbreviations, enzyme commission numbers, electrophoretic conditions, and stain recipes are presented elsewhere [47]. Allozymes were labelled alphabetically and multiple loci, where present, were labelled numerically in order of increasing electrophoretic mobility (e.g. *Acp*^a ^<*Acp*^b^; *Acon-1 *<*Acon-2*).

The genetic affinities of individuals were explored using 'stepwise' Principal Co-ordinates Analysis (PCO), implemented on a pairwise matrix of Rogers' genetic distances. The rationale and methodological details of stepwise PCO are detailed elsewhere [48]. Scatterplots of PCO scores in the first two dimensions were assessed for the presence of discrete clusters of individuals which were diagnosable from all other clusters by the presence of multiple fixed differences (i.e. loci at which the two groups shared no alleles). Separate rounds of PCO were then undertaken individually on these primary groups to assess whether any group harboured additional subgroups which were also diagnosable by multiple fixed differences. Having identified groups of individuals diagnosable from one another by multiple fixed differences, two between-taxon estimates of genetic similarity were calculated: (1) percentage fixed differences (%FD; 1), allowing a cumulative 10% tolerance for any shared alleles, and (2) Nei's unbiased Distance.

### DNA laboratory protocols and phylogenetic analyses

DNA extraction and amplification protocols for *ND2 *and nuclear loci (*RAG-1*, *C-mos*) follow those outlined elsewhere [4,12,28]. Newly-obtained PCR products for this study were sequenced by the Australian Genome Research Facility in Adelaide using an AB3730 DNA Analyzer (Applied Biosystems) and Big Dye chemistry. New sequences were aligned and compared to pre-existing datasets, and translated to check for substitutions leading to stop codons or frameshifts using standard procedures [4,12,28]. Maximum Parsimony (PAUP* vb80) [49], Bayesian Inference (MrBayes v3.1.2) [50] and Maximum Likelihood (RaxML v7.0.4) [51] were used to estimate phylogenetic relationships.

The final *ND2 *alignment consisted of 828 sites. All sequences could be translated into protein with no evidence of misplaced stop codons. Within the genus *Crenadactylus *380 sites were invariable, 32 were variable but not parsimony informative, and 416 were variable and parsimony informative. The final complete nuclear alignment consisted of 2253 sites (1740 *RAG-1 *and 513 *C-mos*) of which 88 sites were variable and 28 were parsimony informative within *Crenadactylus*.

We performed both individual and combined analyses for the mitochondrial and nuclear data. The mitochondrial data were partitioned into first, second and third base pair positions as previous studies using the same gene region and many of the same taxa have demonstrated this significantly improves likelihood [28]. The Akaike information criteria in MrModeltest [52] found the GTR+I+G model to have the highest likelihood for all partitions. For our nuclear alignment we did not partition by gene, (see justification given elsewhere [12]) and compared likelihood and topology for three partitioning strategies (unpartitioned; by codon; 1st with 2nds, 3rds separate). Whereas all strategies returned the same topology, likelihood support for the two partition (1st with 2nds, 3rds separate) strategy was highest. Based on the Akaike Information Criterion we used the GTR+I+G model for 1st and 2nd sites, and the GTR+G model for 3rd sites. Combined mitochondrial and nuclear analyses were partitioned by gene, but otherwise partitioned as per the non-combined analysis. As phylogenetic inference has been shown to be robust to such missing data, especially if it is evenly distributed across divergent lineages [53], the combined dataset included some individuals for which nuclear sequence data were unavailable.

Final Bayesian analyses were run for 5 million generations × 4 chains (one cold and three heated) sampling every 200 generations, with a burn-in of 20% (5,000 trees), leaving 20,000 trees for posterior analysis. In all Bayesian analyses, comparison of parallel runs showed posterior probability convergence (standard deviation <0.01) and likelihood equilibrium, were reached within the burn-in phase. The Maximum Likelihood tree was calculated using the -f d search function in RaxML v7.0.4 and Maximum Likelihood bootstrap support values were calculated using the -f i search function for one thousand replicates. We experimented with both simple and complicated models and found that topology, branch lengths and support values were effectively identical. Maximum Parsimony analyses were performed using heuristic searches with 100 random additions of sequences to identify most parsimonious trees. Bootstrap support values for nodes in MP trees were calculated using 100 pseudo replicates.

### Molecular dating

Divergences dates were estimated using Bayesian dating in BEAST v.1.4 [54]. Dating analyses were performed on three sets of alignment data; *RAG-1 *nuclear data only (nuc), nuclear and mtDNA data combined (comb), and nuc and mtDNA combined with 3rd positions removed from the mtDNA dataset (comb reduced). Mitochondrial data were not analysed alone as the combination of old calibrations and high levels of saturation at this locus would generate significant overestimation of dates [55]. Comparisons between these different analyses focused on variation in both actual and relative date estimates [56], for A) Pygopodoidea, B) Carphodactylidae, C) Pygopodidae, D) Diplodactyidae, E) core Australian Diplodactylidae (as used by Oliver and Sanders [12]), F) crown *Crenadactylus*, and (G-J) major geographically isolated clades within *Crenadactylus *(Table 3, Figure 2A).

Relaxed clock uncorrelated lognormal and GTR+I+G models were applied to all partitions and analyses. Nuclear only dating analyses were run unpartitioned, whereas combined analyses were partitioned into nuclear and mitochondrial data. After multiple initial runs to optimise parameters and priors, final BEAST analyses were run for 10,000,000 generations sampling every 1000 generations using the Yule speciation prior. Adequate sampling and likelihood stability was assessed using TRACER [55]. Two thousand trees (20%) were discarded as burn in. All BEAST runs reached independence and showed no evidence of autocorrelation for all relevant parameters (e.g. branch lengths, topology and clade posteriors).

We used secondary calibrations from two independent studies [11,12] as broad secondary priors; basal divergences among diplodactyloids (mean 71.5 mya, 95% CI 50-90 mya, normal distribution) and a uniform prior at the root of our tree (all geckos 80-150 mya). The latter prior was primarily inserted to provide a broad constraint to ensure analyses never converged on unrealistic dates, and was not meant to explicitly reflect current estimates for the age of this radiation. We experimented with incorporation of a potential calibration within crown Pygopodidae, but while this fossil is clearly a pygopod, its position within the extant radiation is uncertain and it thus does not constrain dates very tightly [57], and its incorporation had negligible effect on date estimates, both within the Pygopodidae and amongst other clades (results not shown).

As an independent check of our inferred date estimates, we estimated rates of mitochondrial evolution within *Crenadactylus *using posterior age estimates from the nuclear and two different combined analyses. A reduced mitochondrial dataset was calibrated with normal priors reflecting the posterior age estimates for the genus, and the mean and range of rates of variation were then estimated using BEAST with settings outlined above.

## Abbreviations

*ND2*: mitochondrial NADH dehydrogenase subunit 2; *RAG-1*: recombination activating gene 1; *C-mos*: Oocyte-maturation factor.

## Authors' contributions

PO and PD conceived the study. PO and MA collected the data. PO and MA wrote the paper. All authors have read and approved the manuscript.

## Supplementary Material

Additional file 1 **Supplementary tables**. **Table S1**: Allozyme frequencies at all loci scored. **Table S2**: Mean intraspecific mtDNA divergences between candidate taxa. **Table S3**: Specimen and sequence details for *Crenadactylus *included in analyses. **Table S4**: Outgroup sequence details. Click here for file

Additional file 2 **Figure S1**. Bayesian tree from combined *RAG1 *and *ND2 *dataset. Click here for file

## References

[B1] Cebellos G Ehrlich P Discoveries of new mammal species and their implications for conservation and ecosystem services Proc Natl Acad Sci USA 2009 106 3841 3846 10.1073/pnas.0812419106 19228946 PMC2656167

[B2] Hebert PDN Penton EH Burns JM Janzen DH Hallwachs W Ten species in one: DNA barcoding reveals cryptic species in the neotropical skipper butterfly *Astraptes fulgerator.* Proc Natl Acad Sci USA 2004 101 14812 14817 10.1073/pnas.0406166101 15465915 PMC522015

[B3] Fouquet A Gilles A Vences M Marty C Blanc M Gemmel NJ Underestimation of species richness in neotropical frogs revealed by mtDNA analyses Plos One 2007 10 e1109 1-10 10.1371/journal.pone.0001109 PMC2040503 17971872

[B4] Oliver PM Doughty P Hutchinson MN Lee MSY Adams A The taxonomic impediment in vertebrates: DNA sequence, allozyme and chromosomal data double estimates of species diversity in a lineage of Australian lizards (*Diplodactylus*, Gekkota) Proc Roy Soc London: B 2009 276 2001 2007 10.1098/rspb.2008.1881 PMC2677245 19324781

[B5] Bickford D Lohman DJ Sodhi NS Ng PKL Meier R Winker K Ingram KK Das I Cryptic species as a window on diversity and conservation Trends Ecol Evol 2007 22 148 55 10.1016/j.tree.2006.11.004 17129636

[B6] Crozier R Genetic diversity and the agony of choice Biol Cons 1992 61 11 15 10.1016/0006-3207(92)91202-4

[B7] Faith DP Phylogenetic diversity and taxonomic priorities for conservation Biol Cons 1992 61 1 10 10.1016/0006-3207(92)91201-3

[B8] Roca AL Bar-Gal G Eizirik E Helgen KM Maria R Springer MS O'Brien SJ Murphy WJ Mesozoic origin for West Indian insectivores Nature 2004 429 649 651 10.1038/nature02597 15190349

[B9] Wake DB Roth G Wake MH On the problem of stasis in organismal evolution J Theor Biol 1983 101 211 224 10.1016/0022-5193(83)90335-1

[B10] Rocha-Olivares A Fleeger JW Foltz DW Decoupling of molecular and morphological evolution in deep lineages of a Meiobenthic Harpactoid Copepod Mol Biol Evol 2001 18 1088 1102 11371597 10.1093/oxfordjournals.molbev.a003880

[B11] Gamble TP Bauer AM Greenbaum E Jackman TR Evidence of Gondwanan vicariance in an ancient clade of gecko lizards J Biogeography 2008 35 88 104

[B12] Oliver PM Sanders K Molecular evidence for multiple lineages with ancient Gondwanan origins in a diverse Australian gecko radiation J Biogeography 2009 36 2044 2055 10.1111/j.1365-2699.2009.02149.x

[B13] Dixon JR Kluge AG A new gekkonid lizard genus from Australia Copeia 1964 174 80 10.2307/1440848

[B14] Storr GM Seven new gekkonid lizards from Western Australia Rec West Aust Mus 1978 7 337 50

[B15] Wilson S Swan G A complete guide to reptiles of Australia 2008 2 New Holland Press, Sydney

[B16] Zamudio KR Greene HW Phylogeography of the bushmaster (*Lachesis muta*: Viperidae): implications for neotropical biogeography, systematics, and conservation Biol J Linn Soc 1997 62 421 442

[B17] Harvey MS Short Range endemism amongst the Australian fauna: some examples from non-marine environments Invertebrate Systematics 2002 16 555 570 10.1071/IS02009

[B18] Solem A McKenzie NL McKenzie NL, Johnston RB, Kendrick PG The composition of land snail assemblages in Kimberley rainforests Kimberley Rainforests of Australia 1991 Sydney: Surrey Beatty and sons 247 263

[B19] Hoskin CJ Australian microhylid frogs (*Cophixalus *and *Austrochaperina*): phylogeny, taxonomy, calls, distributions and breeding biology Aust J Zool 2004 52 237 269 10.1071/ZO03056

[B20] Melville J Swain R Mitochondrial dna-sequence based phylogeny and biogeography of the snow skinks (Squamata: Scincidae: *Niveoscincus) *of Tasmania Herpetologica 2000 56 196 208

[B21] Doughty P Anstis M Price P A new species of *Crinia *(Anura: Myobatrachidae) from the high rainfall zone of the northwest Kimberley, Western Australia Rec West Aust Mus 2009 25 125 144

[B22] Williams SE Bolitho EE Fox S Climate change and Australian tropical rainforests: an impending environmental catastrophe Proc Roy Soc London 2003 270 1887 1892 10.1098/rspb.2003.2464 PMC1691452 14561301

[B23] Horner P Systematics of the snake-eyed skinks, *Cryptoblepharus *Wiegmann (Reptilia: Squamata: Scincidae) - an Australian-based review Rec Mus N Terr 2007 Supp 3 2 98

[B24] Colborn J Crabtree RE Shaklee JB Pfeiler E Bowen BW The evolutionary enigma of bonefishes (*Albula *spp.): cryptic species and ancient divergences in a globally distributed shorefish Evolution 2001 54 807 820 10.1554/0014-3820(2001)055[0807:TEEOBA]2.0.CO;2 11392398

[B25] Lefébure T Douady CJ Gouy M Trontel P Briolays J Gibert J Phylogeography of a subterranean amphipod reveals cryptic diversity and dynamic evolution in extreme environments Mol Ecol 2006 15 1797 1806 16689899 10.1111/j.1365-294X.2006.02888.x

[B26] Martin AP Palumbi SR Body size, metabolic rate, generation time, and the molecular clock Proc Natl Acad Sci USA 1993 90 4087 4091 10.1073/pnas.90.9.4087 8483925 PMC46451

[B27] Couper P Sadlier R Shea GM Worthington Wilmer J A reassessment of *Salturius swaini *(Lacertilia: Diplodactylidae) in southeastern Queensland and New South Wales; Two new taxa, phylogeny, biogeography and conservation Rec Aust Mus 2008 60 87 118 10.3853/j.0067-1975.60.2008.1492

[B28] Oliver PM Hugall A Adams M Cooper SJB Hutchinson M Genetic elucidation of ancient and cryptic diversity in a group of Australian geckos: the *Diplodactylus vittatus *complex Mol Phyl Evol 2007 44 77 88 10.1016/j.ympev.2007.02.002 17467299

[B29] Pepper M Doughty P Keogh JS Molecular systematics and phylogeography of the Australian *Diplodactylus stenodactylus *(Gekkota: Reptilia) species-group based on mitochondrial and nuclear genes reveals an ancient split between Pilbara and non-Pilbara *D. stenodactylus* Mol Phyl Evol 2006 41 539 555 10.1016/j.ympev.2006.05.028 16843684

[B30] Kozak KH Weisrock DW Larson A Rapid lineage accumulation in a non-adaptive radiation: phylogenetic analysis of diversification rates in eastern North American woodland salamanders Proc Roy Soc London: B 2006 273 539 546 10.1098/rspb.2005.3326 PMC1560065 16537124

[B31] Sanders KL Lee MSY Molecular evidence for a rapid late-Miocene radiation of Australasian venomous snakes (Elapidae, Colubroidea) Mol Phyl Evol 2008 46 1165 1173 10.1016/j.ympev.2007.11.013 18222097

[B32] Hugall AF Foster R Hutchinson M Lee MSY Phylogeny of Australasian agamid lizards based on nuclear and mitochondrial genes: implications for morphological evolution and biogeography Biol J Linn Soc 2008 93 343 358 10.1111/j.1095-8312.2007.00911.x

[B33] Meredith RW Westerman M Springer MS A phylogeny and timescale for the living genera of kangaroos and kin (Macropodiformes: Marsupialia) based on nuclear DNA sequences Aust J Zool 2008 56 395 410 10.1071/ZO08044

[B34] Rowe KC Reno ML Richmond DM Adkins RM Steppan SJ Pliocene colonization and adaptive radiations in Australia and New guinea (Sahul): Multilocus systematics of the old endemic rodents (Muroidea: Murinae) Mol Phy Evol 2008 47 84 101 10.1016/j.ympev.2008.01.001 18313945

[B35] Avise JC Aquadro CF A comparative study of genetic distances in the vertebrates Evol Biol 1982 15 151 185

[B36] Georges A Adams M A phylogeny for the Australian chelid turtles based on allozyme electrophoresis Aust J Zool 1992 40 453 476 10.1071/ZO9920453

[B37] Barker FK Cibois A Schikler P Feinstein J Cracraft J Phylogeny and diversification of the largest avian radiation Proc Natl Acad Sci USA 2004 111 11040 45 10.1073/pnas.0401892101 PMC503738 15263073

[B38] Meredith RW Westerman M Springer MS A phylogeny of the Diprodontia (Marsupialia) based on sequences for five nuclear genes Mol Phyl Evol 2009 51 554 571 10.1016/j.ympev.2009.02.009 19249373

[B39] Roelants K Gower DJ Wilkinson M Loader SP Biju SD Guillaume K Moriau L Bossuyt F Global patterns of diversification in the history of modern amphibians Proc Natl Acad Sci USA 2007 104 887 892 10.1073/pnas.0608378104 17213318 PMC1783409

[B40] Sauquet H Weston PH Anderson CL Barker NP Cantrill DJ Mast AR Savolainen V Contrasted patterns of hyperdiversification in Mediterranean hotspots Proc Natl Acad Sci USA 2009 106 221 225 10.1073/pnas.0805607106 19116275 PMC2629191

[B41] Cook LG Crisp MD Not so ancient: the extent crown group of *Nothofagus *represents a post-Gondwanan radiation Proc Roy Soc Lond B 2004 272 2535 2544 10.1098/rspb.2005.3219 PMC1599775 16271980

[B42] Vitt LJ Sartorius SS Avila-Pires TCS Zani PA Espósito MC Small in a big world: Ecology of leaf-litter geckos in new world tropical forests Herp Monographs 2005 19 137 152 10.1655/0733-1347(2005)019[0137:SIABWE]2.0.CO;2

[B43] Byrne M Yeates DK Joseph L Kearney M Bowler J Williams MAJ Cooper S Donnellan SC Keogh JS Leys R Melville J Murphy DJ Porch N Wyrwoll KH Birth of a biome: Insights into the assembly and maintenance of the Australian arid zone biota Mol Ecol 2008 17 4398 4417 10.1111/j.1365-294X.2008.03899.x 18761619

[B44] Leys R Watts C Cooper SJB Humphreys WF Evolution of subterranean diving beetles (Coleoptra: Dytiscidae: Hydroporini, Bidessini) in the arid zone Evolution 2003 57 2819 2834 14761060 10.1111/j.0014-3820.2003.tb01523.x

[B45] Dayrat B Towards integrative taxonomy Biol J Linn Soc 2005 85 407 415 10.1111/j.1095-8312.2005.00503.x

[B46] Padial JM Miralles A De la Riva I Vences M The integrative future of taxonomy Frontiers in Zoology 2010 7 16 10.1186/1742-9994-7-16 20500846 PMC2890416

[B47] Richardson BJ Baverstock PR Adams MA Allozyme electrophoresis: A handbook for animal systematics and population studies 1986 Sydney: Academic press

[B48] Horner P Adams M A molecular systematic assessment of species boundaries in Australian *Cryptoblepharus *(Reptilia: Squamata: Scincidae) - a case study for the combined use of allozymes and morphology to explore cryptic biodiversity Rec Mus N Terr 2009 Supp 3 1 19

[B49] Swofford DL PAUP*: Phylogenetic Analysis using Parsimony * (and other methods). Version 4 2000 Sunderland, MA: Sinauer

[B50] Huelsenbeck JP Ronquist F MRBAYES: Bayesian inference of phylogeny Bioinformatics 2001 17 754 755 10.1093/bioinformatics/17.8.754 11524383

[B51] Stamatakis A RaxML-VI-HPC: Maximum Likelihood-based Phylogenetic analyses with thousands of taxa and mixed models Bioinformatics 2006 22 2688 2690 10.1093/bioinformatics/btl446 16928733

[B52] Nylander JAA MrModeltest 2004 http://www.abc.se/~nylander/

[B53] Wiens JJ Fetzner JW Parkinson CL Reeder TW Hylid frog phylogeny and sampling strategies for speciose clades Syst Biol 2005 54 719 748 10.1080/10635150500234534 16243760

[B54] Drummond AJ Rambaut A BEAST v 1.4 2006 http://beast.bio.ed.ac.uk/

[B55] Loader SP Pisani D Cotton JA Gower DJ Day JJ Wilkinson M Relative timescales reveal multiple origins of parallel disjunct distributions of African caecilian amphibians Biol Letters 2007 3 505 508 10.1098/rsbl.2007.0266 PMC2396187 17609171

[B56] Jansa SA Barker FK Heaney LR The pattern and timing of diversification of Phillipene endemic rodents: evidence from mitochondrial and nuclear gene sequences Syst Biol 2006 55 73 88 10.1080/10635150500431254 16507525

[B57] Lee MSY Oliver P Hutchinson MN Phylogenetic uncertainty and molecular clock calibrations: a case study of legless lizards (Pygopodidae: Gekkota) Mol Phyl Evol 2009 50 661 666 10.1016/j.ympev.2008.11.024 19111935

