# Supplementary material for: Molecular evidence for ten species and Oligo-Miocene vicariance within a nominal Australian gecko species (Crenadactylus ocellatus, Diplodactylidae)
Source: BMC Evol Biol. 2010 Dec 15;10:386. doi: 10.1186/1471-2148-10-386 (PMC3018458; doi:10.1186/1471-2148-10-386)
Supplement: Additional file 1 — Supplementary tables. Table S1: Allozyme frequencies at all loci scored. Table S2: Mean intraspecific mtDNA divergences between candidate taxa. Table S3: Specimen and sequence details for Crenadactylus included in analyses. Table S4: Outgroup sequence details. [file 1471-2148-10-386-S1.DOC]

## Table S1. Allozyme frequencies for 10 candidate species of *Crenadactylus* at 37 variable loci. For polymorphic loci, the frequencies of all but the rarer/rarest alleles are expressed as percentages and shown as superscripts (allowing the frequency of each rare allele to be calculated by subtraction from 100%). Alleles joined without being separated by a comma all shared the frequency indicated. A dash indicates no genotypes were assignable at this locus. The maximum number of individuals sampled for each taxon is shown in brackets. Invariant loci: *Ak-1*, *Enol*, *Lap*, *Npdk-1*, and *Pgam*.

| **Locus** | **South West**  (35) | **Carnarv Basin**  (16) | **Cape Range**  (4) | **Pilbara**  (15) | **Kimb**  **A**  (1) | **Kimb**  **B**  (7) | **Kimb**  **C**  (1) | **Kimb**  **D**  (1) | **Kimb**  **E**  (3) | **Central Ranges**  (11) |
| --- | --- | --- | --- | --- | --- | --- | --- | --- | --- | --- |
| *Acon-1* | d^99^,b | a | d^75^,a^13^,c | a | d | d | e | f | e | a |
| *Acon-2* | h^90^,e^7^,k^2^,i | l^63^,m^18^,  k^13^,g | f^83^,h | f^93^,a | d | h^43^,f^36^,  b^14^,c | f | f | f | j^91^,f |
| *Acp* | d^97^,b | d | c | e^87^,d | d | c | e | b^50^,d | e | d^95^,a |
| *Acyc* | b | c^80^,b | d | b | b | a | b | b | b | a |
| *Adh-1* | b^93^,c^5^,a | b | d^75^,b | b^79^,e | b | b | b | b | b | b |
| *Adh-2* | d^96^,g | d^97^,a | d | c^73^,b^17^,e^7^,f | a^50^,b | d^93^,b | d | a | a | a |
| *Ak-2* | a^97^,b^3^ | a | a^75^,c^25^ | a | a | a | a | a | a | a |
| *Dia* | f^91^,c^4^,h^2^,  abd^1^ | g^53^,d^33^,  e^7^,h^4^,f | f | h^47^,c^37^,  f^7^,g^6^,i | g | h | h | g | g^67^,h | f^95^,c |
| *Est* | c^69^,b^20^,e^9^,a | c | e | e | e | e^50^,g^29^,f | c | c | c | c^70^,d^25^,e |
| *Fdp* | b^79^,a^16^,c | b | a | a | a | a | a | a | a | b^82^,a |
| *Fum* | a | e | c^75^,d | c | c^50^,g | c | c | e | c^83^,f | c^95^,b |
| *Gapd* | a^98^,b^2^ | a | a^88^,b^12^ | a | a | a | a | a | a | a |
| *Glo* | b | b^91^,a | b | b^96^,d | b | b^93^,c | b | b | b | b |
| *Got-1* | b^54^,e^31^,  c^14^,a | e | e | e^60^,  b^37^,g | e | e^93^,d | d | e | e | e^95^,f |
| *Got-2* | b^97^,a^3^ | b | b | c | d | d | d | d | d | b |
| *Gpd-1* | d^98^,a | d^90^,f | d^67^,e^17^,g | b | b | b^93^,e | b | b | b | c |
| *Gpd-2* | b^96^,c | b^45^,c^42^,a | c | b | e | c^70^,d | c | b | a^50^,c | b |
| *Gpi* | b | b | b^87^,a | b | b | b | b | b | b | b |
| *Gsr* | h^31^,k^17^,l^16^,n^14^,m^11^,j^9^,i | h^31^,f^25^,  j^25^,c^13^,g | h | g^93^,i^4^,d | a | a^93^,b | c | b | b | c^95^,e |
| *Idh* | e^97^,d | d | b | c | c^50^,g | c^93^,f | a | c | c | e |
| *Ldh-1* | a | a | a | a | a | a | a | a | a | a^95^,b |
| *Ldh-2* | a | b | a | a | b | b | a | a | a | a |
| *Mdh-1* | h^60^,d^27^,  g^7^,b^3^,a | e | d | f | f | f | f | f | d^67^,f | f^95^,c |
| *Mdh-2* | e^91^,c | d | e^88^,f | e^93^,b | e | e | e | e | e | e^50^,c^45^,a |
| *Mpi* | d^91^,f | d^87^,e | d | c^93^,e^4^,b | c | c^86^,a | c | b | b | d^86^,f^9^,e |
| *Ndpk-2* | c^99^,a | c | a | c | c | c^79^,a | a | a | b^83^,a | c |
| *Ntak* | b^79^,c^19^,af^1^ | b | b^75^,d | e | e | e | e | e | e | g^91^,f^5^,h |
| *PepA* | b^98^,d | b | b^83^,e | c | e | e | e | e | e | e^95^,a |
| *PepB* | c^94^,e^4^,a | c | c | c | f | d^64^,f^29^,b | f | f | f | c |
| *6Pgd* | e^98^,c | e^83^,b | e | d^86^,a^11^,f | e | e | g | e | b^50^,e | e^95^,g |
| *Pgk* | c^98^,a | c | c^75^,d | b | b^50^,c | b | c | c | c | b |
| *Pgm-1* | d^70^,a^22^,f^7^,b | e^87^,c | e | h^68^,i^25^,j | e | e^64^,f^22^,b | f | d^50^,e | d | h^68^,g |
| *Pgm-2* | b^99^,a | b | b | b | b | b^86^,c | b | b | b | b |
| *Sod* | e^99^,d | e | e | c^97^,a | e | c^93^,b | e | e | e | e^95^,d |
| *Sordh* | b | c^81^,d | e | b^96^,a | - | b | b | - | a | a |
| *Tpi* | b^97^,d | b | b | a | c | c | c | c | c | c |
| *Ugpp* | a | a | a | a | a | a | a | a | a | b |

Table S2. Uncorrected and corrected (GTR+I+G) genetic distances within ten candidate species of *Crenadactylus*, calculated from 828 bp of ND2 data.

|  | N | Uncorrected | corrected |
| --- | --- | --- | --- |
| **1. South-west** | 7 | 0.002 (0.000-0.005) | 0.002 (0.000-0.004) |
| **2. Cape Range** | 4 | 0.001 (0.000-0.001) | 0.000 (0.000-0.001) |
| **3. Carnarvon** | 10 | 0.013 (0.000-0.022) | 0.012 (0.000-0.020) |
| **4. Pilbara** | 10 | 0.062 (0.002-0.097) | 0.071 (0.000-0.116) |
| **5. Central Ranges** | 11 | 0.056 (0.002-0.079) | 0.059 (0.002-0.090) |
| **6. Kimberly A** | 1 | NA | NA |
| **7. Kimberly B** | 1 | NA | NA |
| **8. Kimberly C** | 2 | NA | NA |
| **9. Kimberly D** | 1 | NA | NA |
| **10. Kimberly E** | 7 | 0.021 (0.001-0.034) | 0.019 (0.003-0.032) |

Table S3. Specimen, locality and Genbank details for all individuals of *Crenadactylus 'ocellatus'* sequenced for either ND2, RAG1 or C-mos, and/or included in allozyme analyses

| Taxon | LOCALITY | Exnum | STATE | ND2 | RAG1 | C-mos | allozymes | LAT | LONG |
| --- | --- | --- | --- | --- | --- | --- | --- | --- | --- |
| South West | Norseman | DV355 | WA | _ | _ | _ | x | 320923S | 1214424E |
| South West | Walganna Rock | DV361 | WA | HQ288428 | _ | _ | x | 272400S | 1172800E |
| South West | 4km N Ravensthorpe | DV379 | WA | _ | _ | _ | x | 333200S | 1200300E |
| South West | Yorkrakine Rock | DV380 | WA | HQ288430 | _ | _ | x | 312600S | 1173100E |
| South West | Bindoon Military Training Area | DV381 | WA | HQ288428 | _ | _ | x | 311344S | 1161738E |
| South West | West Wallabi Island | DV382 | WA | _ | _ | _ | x | 282900S | 1134100E |
| South West | West Wallabi Island | DV421 | WA | _ | _ | _ | x | 282900S | 1134100E |
| South West | Spalding Park,Geraldton | DV423 | WA | _ | _ | _ | x | 284600S | 1143700E |
| South West | Murray Island | DV424 | WA | _ | _ | _ | x | 285347S | 1135352E |
| South West | Irwin R | DV425 | WA | _ | _ | _ | x | 285800S | 1152900E |
| South West | Bungalbin Woodland Camp | DV426 | WA | _ | _ | _ | x | 301812S | 1194346E |
| South West | Esscape Island | DV427 | WA | _ | _ | _ | x | 302002S | 1145904E |
| South West | 55km NNW Norseman | DV428 | WA | _ | _ | _ | x | 314600S | 1214000E |
| South West | Old Badgingara Townsite | DV429 | WA | _ | _ | _ | x | 302500S | 1153400E |
| South West | North Cervantes Island | DV430 | WA | _ | _ | _ | x | 303200S | 1150300E |
| South West | Bindoon Military Training Area | DV431 | WA | _ | _ | _ | x | 311553S | 1161519E |
| South West | Eglinton | DV432 | WA | _ | _ | _ | x | 313900S | 1154100E |
| South West | Neerabup | DV433 | WA | _ | _ | _ | x | 314000S | 1154500E |
| South West | Darling Ra. Behind Brigadon Estate | DV434 | WA | _ | _ | _ | x | 314600S | 1160700E |
| South West | Darlington | DV435 | WA | _ | _ | _ | x | 315500S | 1160400E |
| South West | 7KM NE Kellerberrin | DV437 | WA | _ | _ | _ | x | 313600S | 1174600E |
| South West | Boodaring Rock | DV438 | WA | _ | _ | _ | x | 313621S | 1194827E |
| South West | Yellowdine | DV439 | WA | _ | _ | _ | x | 311800S | 1193900E |
| South West | Dedari | DV440 | WA | _ | _ | _ | x | 310500S | 1204500E |
| South West | Nr Carracarrup Pool | DV443 | WA | _ | _ | _ | x | 334425S | 1195835E |
| South West | Kordinrup Dam, 6KM ESE Ravensthorpe | DV444 | WA | _ | _ | _ | x | 333700S | 1200700E |
| South West | Spalding Park,Geraldton | DV594 | WA | HQ288431 | _ | _ | x | 284600S | 1143700E |
| South West | Mcdermid Rock | DV596 | WA | HQ288432 | _ | _ | x | 320100S | 1204400E |
| South West | Ravensthorpe | DV598 | WA | HQ288433 | HQ288477 | _ | x | 333500S | 1200200E |
| South West | Dryandra | DV601 | WA | HQ288434 | _ | _ | x | 324702S | 1165514E |
| South West | Murray Island | NA | WA | _ | _ | _ | x | 285347S | 1135352E |
| Pilbara | Burrup Peninsula | DV288 | WA | HQ288452 | _ | _ | x | 203645S | 1164737E |
| Pilbara | Deepdale oustation, Robe River | DV362 | WA | HQ288458 | _ | _ | x | 214300S | 1161100E |
| Pilbara | 80 km s Telfer | DV363 | WA | _ | _ | _ | x | 222000S | 1220500E |
| Pilbara | 20KM WSW Pannawonica | DV399 | WA | _ | _ | _ | x | 214400S | 1161000E |
| Pilbara | 5km South Mount Tom Price Mine | DV400 | WA | HQ288453 | _ | _ | x | 224834S | 1174640E |
| Pilbara | 5km South Mount Tom Price Mine | DV401 | WA | HQ288454 | _ | _ | x | 224834S | 1174640E |
| Pilbara | Hope Downs | DV402 | WA | HQ288455 | _ | _ | x | 225800S | 1190700E |
| Pilbara | Burrup Peninsula | DV403 | WA | HQ288449 | _ | _ | x | 203645S | 1164737E |
| Pilbara | Burrup Peninsula | DV404 | WA | HQ288451 | _ | _ | x | 203645S | 1164737E |
| Pilbara | Burrup Peninsula | DV405 | WA | HQ288450 | HQ132627 | _ | x | 203534S | 1164758E |
| Pilbara | 58 KM ESE Meentheena Outcamp | DV446 | WA | HQ288456 | _ | _ | x | 22535S | 118.977E |
| Pilbara | 26 KM WSW Mt Marsh | DV447 | WA | HQ288457 | _ | _ | x | 213219S | 121.002E |
| Pilbara | Burrup Peninsula | NA | WA | _ | _ | _ | x | 203534S | 1164758E |
| Pilbara | Burrup Peninsula | NA | WA | _ | _ | _ | x | 203534S | 1164758E |
| Pilbara | Burrup Peninsula | NA | WA | _ | _ | _ | x | 203534S | 1164758E |
| Kimberleys E | Koolan Island | DV365 | WA | HQ288469 | HQ288480 | _ | x | 160718S | 1234312E |
| Kimberleys E | Koolan Island | DV406 | WA | _ | _ | _ | x | 160821S | 1234453E |
| Kimberleys E | Koolan Island | DV407 | WA | HQ288468 | _ | _ | x | 160814S | 1234529E |
| Kimberleys D | Mitchell Falls | DV285 | WA | HQ288470 | FJ855458 | FJ855479 | x | 144900S | 1254100E |
| Kimberleys C | Augustus Is (NE Corner) | DV366 | WA | HQ288466 | HQ288481 | _ | x | 152700S | 1243800E |
| Kimberleys B | Bream Gorge-Osmond Valley | DV286 | WA | HQ288475 | _ | _ | x | 171500S | 1281800E |
| Kimberleys B | Calico Spring Mabel Downs Stn | DV367 | WA | HQ288474 | _ | _ | x | 171700S | 1281100E |
| Kimberleys B | 25 km se Kununurra | DV368 | WA | HQ288473 | HQ151002 | _ | x | 155600S | 1285400E |
| Kimberleys B | Bream Gorge-Osmond Valley | DV408 | WA | _ | _ | _ | x | 171500S | 1281800E |
| Kimberleys B | Mount Parker | DV409 | WA | _ | _ | _ | x | 171004S | 1281823E |
| Kimberleys B | 25 km se Kununurra | DV410 | WA | HQ288471 | _ | _ | x | 155600S | 1285400E |
| Kimberleys B | 25 km se Kununurra | DV411 | WA | HQ288472 | _ | _ | x | 155600 | 1285400E |
| Kimberleys A | 24 Km N Tunnel Creek | DV364 | WA | HQ288467 | HQ53890 | _ | x | 172841S | 1250118E |
| Central Ranges | 10km S of Barrow Creek | NA | NT | AY369016 | AY662627 | FJ071641 | _ | 213800S | 1335300E |
| Central Ranges | 1.9k SW Sentinel Hill | DV283 | SA | HQ288465 | _ | _ | _ | 260533S | 1322605E |
| Central Ranges | 38k ESE Amata | DV369 | SA | HQ288463 | _ | _ | x | 261714S | 1312930E |
| Central Ranges | Bagot Ck Watarrka NP NT | DV370 | NT | HQ288464 | _ | _ | x | 242200S | 1314800E |
| Central Ranges | 1.9k SW Sentinel Hill | DV412 | SA | HQ288459 | _ | _ | x | 260533S | 1322605E |
| Central Ranges | Lawrence Gorge | DV413 | NT | HQ288460 | _ | _ | x | 240100S | 1332400E |
| Central Ranges | Ellery Creek | DV414 | NT | _ | _ | _ | x | 235000S | 1325800E |
| Central Ranges | 38k ESE Amata | DV415 | SA | X | _ | _ | x | 261714S | 1312930E |
| Central Ranges | 11.2k SW Sentinel Hill | DV416 | SA | X | _ | _ | x | 260828S | 1322133E |
| Central Ranges | 4k SSW Mt Cuthbert | DV417 | SA | _ | _ | _ | x | 260809S | 1320360E |
| Central Ranges | 2.5k SW Womikata Bore | DV418 | SA | _ | _ | _ | x | 260641S | 1320759E |
| Central Ranges | Lawrence Gorge | DV419 | NT | HQ288461 | _ | _ | x | 240100S | 1332400E |
| Central Ranges | 36k W junct Namatjira/Larapinta Drv | DV420 | NT | HQ288462 | _ | _ | x | 234600S | 1331000E |
| Carnarvon | False Entrance Well | DV289 | WA | HQ288446 | FJ855457 | FJ571641 | x | 262300S | 1131900E |
| Carnarvon | Kalbarri | DV357 | WA | _ | _ | _ | x | 274200S | 1141000E |
| Carnarvon | Carnarvon Basin | DV358 | WA | HQ288447 | _ | _ | x | 271541S | 1140148E |
| Carnarvon | 70k S Exmouth | DV359 | WA | HQ288443 | _ | _ | x | 223500S | 1140700E |
| Carnarvon | East Yuna Nature Reserve | DV383 | WA | _ | _ | _ | x | 282800S | 1151300E |
| Carnarvon | 10k NW Wandina HS | DV384 | WA | _ | _ | _ | x | 275600S | 1153300E |
| Carnarvon | Kalbarri N.P. | DV385 | WA | _ | _ | _ | x | 275200S | 1141000E |
| Carnarvon | Kalbarri N.P. | DV386 | WA | _ | _ | _ | x | 274200S | 1141300E |
| Carnarvon | Carnarvon Basin | DV387 | WA | HQ288444 | _ | _ | x | 27249S | 1143423E |
| Carnarvon | False Entrance Well | DV388 | WA | HQ288448 | _ | _ | x | 262300S | 1131900E |
| Carnarvon | False Entrance Well | DV389 | WA | HQ288445 | _ | _ | x | 262300S | 1131900E |
| Carnarvon | Carnarvon Basin, WA -sector CU6 | DV390 | WA | _ | _ | _ | x | 241818S | 1132645E |
| Carnarvon | 5KM s Quobba Homestead | DV391 | WA | HQ288441 | _ | _ | x | 242535S | 1132410E |
| Carnarvon | Red Bluff | DV392 | WA | HQ288439 | _ | _ | x | 240024S | 1132747E |
| Carnarvon | Warroora Station | DV393 | WA | HQ288440 | _ | _ | x | 233900S | 1134800E |
| Carnarvon | Bullara HS, WA | DV394 | WA | _ | _ | _ | x | 224100S | 1140200E |
| Carnarvon | 4k W Bullara HS | DV395 | WA | _ | _ | _ | x | 224100S | 1140200E |
| Carnarvon | 70k S Exmouth | DV396 | WA | HQ288442 | _ | _ | x | 223500S | 1140700E |
| Carnarvon | False Entrance Well | NA | WA | _ | _ | _ | x | 262300S | 1131900E |
| Cape Range | Shothole Canyon Cape Range NP | DV397 | WA | HQ288437 | _ | _ | x | 220300S | 1140100E |
| Cape Range | Shothole Canyon Cape Range NP | DV398 | WA | HQ288438 | _ | _ | x | 220300S | 1140100E |
| Cape Range | Vlaming Head, WA | DV595 | WA | HQ288436 | _ | _ | x | 215000S | 1140500E |
| Cape Range | Shothole Canyon Cape Range NP | DV599 | WA | HQ288435 | HQ288476 | _ | x | 220300S | 1140100E |

Table S4. Specimen and sequence details for species used as outgroups in phylogenetic and molecular analyses.

| **Taxon** | **Specimen** | **Locality** | *RAG-1* | *c-mos* | ***ND2*** |
| --- | --- | --- | --- | --- | --- |
| **Carphodactylids** |  |  |  |  |  |
| *Carphodactylus laevis* | QMJ8944 | Lake Barrine, Qld, Australia | FJ855442 | AF039467 | AY369017 |
| *Nephurus milii* | SAMA R38006 | 17 km SE Burra, South Australia | FJ571622 | FJ571637 | HQ288423 |
| *Nephurus stellatus* | SAMA R36563 | 19.3 km NE Courtabie, South Australia | FJ855446 | FJ855466 | HQ288424 |
| *Nephrurus asper* | SAMAR55649 | 10 km W Isaac R, Qld, Australia | FJ855445 | FJ855465 | HQ288422 |
| *Phyllurus platurus* | ABTC51012 | Bents Basin, Sydney, Australia | FJ855443 | _ | HQ288421 |
| *Phyllurus platurus* | NA | NA | _ | AY172942 | _ |
| *Saltuarius swaini* | SAMAR29204 | Wiangaree, NSW, Australia | FJ855444 | FJ855464 | AY369023 |
| **Diplodactylids** |  |  |  |  |  |
| *Bavayia sauvagei* | AMSR125814 | Mare Island, New Caledonia. | FJ855448 | FJ855468 | _ |
| *Diplodactylus granariensis* | WAMR127572 | Goongarrie, Western Australia | FJ855452 | FJ855473 | _ |
| *Diplodactylus granariensis* | **WAMRxxxxxx** | Mt Jackson, Western Australia | _ | _ | EF532870 |
| *Diplodactylus tessellatus* | SAMAR41130 | Nr Stuart Hwy, South Australia | FJ571624 | FJ571639 | AY134607 |
| *Lucasium byrnei* | SAMA R52296 | Camel Yard Spring, South Australia | FJ855453 | FJ855474 | EF681801 |
| *Luscasium stenodactylum* | NTMR26116 | Mann River, Northern Territory | FJ855454 | FJ855475 | HQ288427 |
| *Oedura marmorata* | SAMAR34209 | Lawn Hill NP, Qld, Australia | FJ571623 | FJ571638 | AY369015 |
| *Oedura reticulata* | SAMA R23035 | 73 km E. Norseman, Western Australia | FJ855450 | FJ855471 | EF681803 |
| *Oedura rhombifer* | SAMA R34513 | Townsville area, Qld, Australia | FJ855451 | FJ855472 | HQ288426 |
| *Pseudothecadactylus australis* | QMJ57120 | Heathlands, Qld, Australia | FJ855449 | FJ855470 | HQ288425 |
| *Pseudothecadactylus lindneri* | AMS90915 | Liverpool R, NT, Australia | AY662626 | FJ855469 | AY369024 |
| *Rhychoedura ornata* | SAMAR36873 | Mern Merna Station, South Australia | FJ855455 | FJ855476 | _ |
| *Rhychoedura ornata* | ANWCR6141 | Native Gap, Stuart Hwy, Northern Territory | _ | _ | AY369014 |
| *Strophurus intermedius* | SAMAR28963 | Gawler Ranges, South Australia | FJ571625 | FJ571640 | _ |
| *Strophurus intermedius* | SAMAR22768 | Uro Bluff, South Australia | _ | _ | AY369001 |
| *Strophurus jeanae* | SAMAR53984 | 11 km S. of Wycliffe Well | FJ855456 | FJ855477 | _ |
| **Pygopodids** |  |  |  |  |  |
| *Aprasia inaurita* | SAMAR40729 | 2 km E of Burra, South Australia | FJ571632 | FJ571646 | _ |
| *Aprasia inaurita* | SAMAR47087 | ST Peters Island | _ | _ | AY134574 |
| *Delma australis* | SAMAR22784 | Mt Remarkable NP, South Australia | FJ571633 | FJ571647 | AY134582 |
| *Delma molleri* | SAMAR23137 | Mt Remarkable NP, South Australia | FJ571635 | FJ571649 | AY134593 |
| *Lialis jicari* | TNHC59426 | NA | AY662628 | _ | _ |
| *Lialis jicari* | NA | Irian Jaya | _ | AY134564 | AY134600 |
| *Ophidiocephalus taeniatus* | SAMAR44653 | Todmorden Stn, South Australia | FJ571630 | FJ571645 | AY134601 |
| *Pletholax gracilis* | WAM R104374 | Victoria Park, Western Australia | FJ571631 | _ | AY134602 |
| *Pletholax gracilis* | WBJ-2483 | Lesueur National Park, Western Australia | _ | AY134566 | _ |
| *Paradelma orientalis* | QMJ56089 | 20 km N Capella, Qld, Australia | FJ571626 | FJ571642 | AY134605 |
| *Pygopus lepidopodus* | WAM R90378 | Walpole-Nornalup NP, Western Australia | FJ571627 | FJ571643 | _ |
| *Pygopus lepidopodus* | WBJ-1206 | Lesueur National Park, Western Australia | _ | _ | AY134603 |
| **Other gekkonids** |  |  |  |  |  |
| *Gehyra variegata* | SAMAR54022 | Brunette Downs, NT, Australia | FJ855439 | FJ855460 | _ |
| *Gehyra variegata* | ANWCR6138 | Old Andado Homestead, Northern Territory | _ | _ | AY369026 |
| *Gekko gekko* | MVZ215314 | NA | AY662625 | _ | AF114249 |
| *Gekko gekko* | FMNH258696 | NA | _ | AY444028 | _ |
| *Teratoscincus przewalski* | CAS171010 | South Gobi Desert Mongolia | AY662624 | AY662569 | U71326 |
| *Sphaerodactylus shreveri* | SBH194572 | Haiti | AY662623 | AY662547 | AY662547 |
