# Supplementary material for: Molecular evidence for ten species and Oligo-Miocene vicariance within a nominal Australian gecko species (Crenadactylus ocellatus, Diplodactylidae)
Source: BMC Evol Biol. 2010 Dec 15;10:386. doi: 10.1186/1471-2148-10-386 (PMC3018458; doi:10.1186/1471-2148-10-386)
Supplement: Additional file 2 — Figure S1. Bayesian tree from combined RAG1 and ND2 dataset. [file 1471-2148-10-386-S2.DOC]

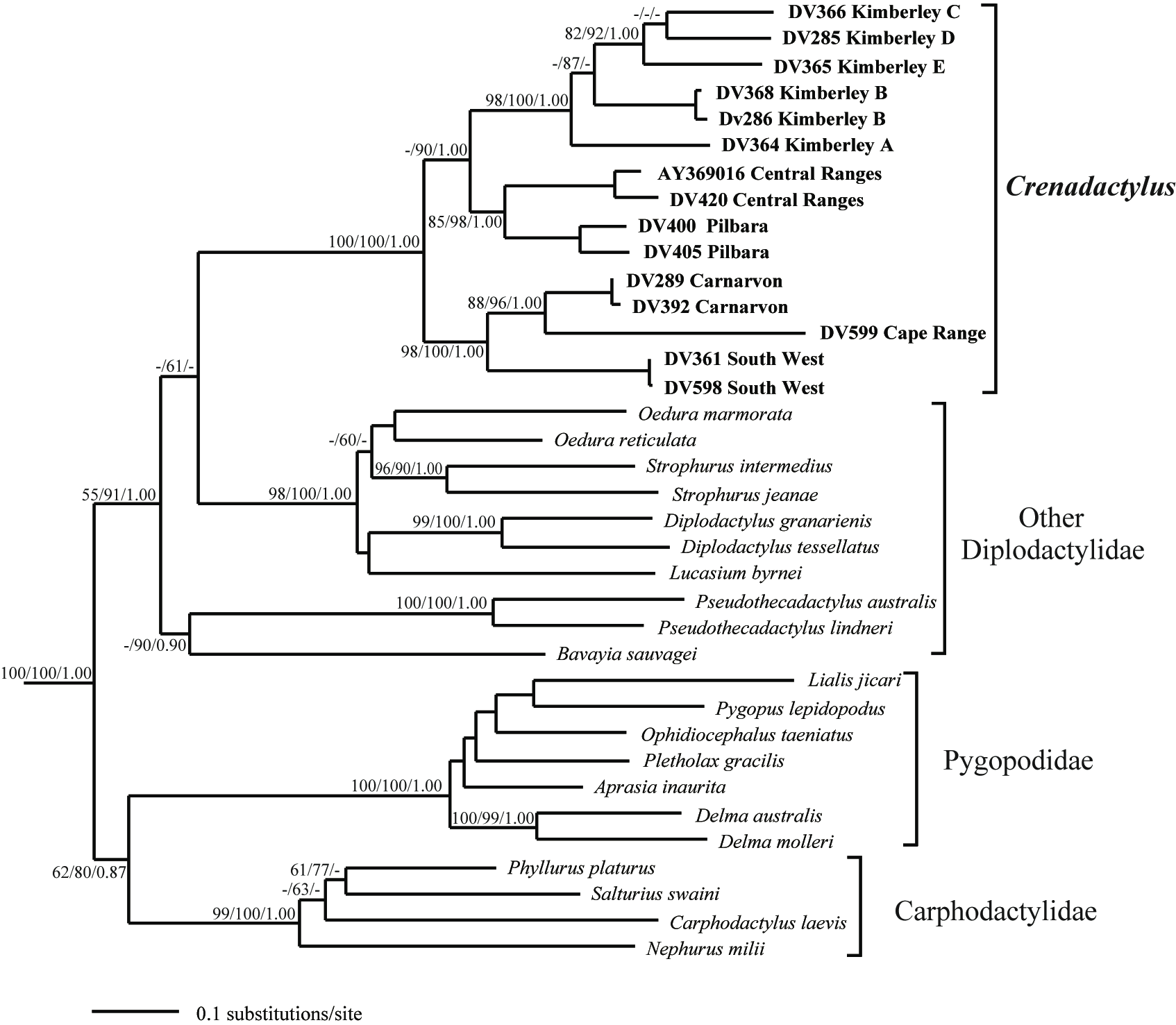


Figure S1. Representative estimate of phylogenetic relationships between 10 candidate species confounded within *Crenadactylus 'ocellatus*' based on combined analysis of 978bp RAG1 and 828bp ND2 for a subset of ingroup specimens spanning major divergences. Consensus phylogram of 20,000 trees from 5 million generation bayesian analyses with a burnin of 20%, support values at major nodes are respectively maximum parsimony (PAUP), maximum likelihood (RaxML) and Bayesian posterior probabilities (MrBayes). See methods and materials for further details of analyses. All analyses supported the same relationships between the major geographically isolated lineages of *Crenadactylus*.
